# Exploring the relationship between caffeine metabolism-related *CYP1A2* rs762551 polymorphism and team sport athlete status and training adaptations

**DOI:** 10.1007/s11033-024-09800-2

**Published:** 2024-07-23

**Authors:** Hasan H. Kazan, Celal Bulgay, Ercan Zorba, Metin Dalip, Halil İ. Ceylan, Ekaterina A. Semenova, Andrey K. Larin, Nikolay A. Kulemin, Edward V. Generozov, Ildus I. Ahmetov, Mesut Cerit

**Affiliations:** 1 Department of Medical Biology, Gulhane Faculty of Medicine, University of Health Sciences, Ankara, 06010 Türkiye; 2 Sports Science Faculty, Bingol University, Bingol, 12000 Türkiye; 3 Faculty Faculty of Sport Sciences, Mugla Sıtkı Kocman University, Muğla, 48000 Türkiye; 4 Faculty of Physical Culture and Health, University in Tetovo, Tetova, 1200 Republic of North Macedonia; 5 Physical Education and Sports Teaching Department, Kazim Karabekir Faculty of Education, Ataturk University, Erzurum, 25240 Türkiye; 6 Department of Molecular Biology and Genetics, Lopukhin Federal Research and Clinical Center of Physical-Chemical Medicine of Federal Medical Biological Agency, Moscow, 119435 Russia; 7 Research Institute of Physical Culture and Sport, Volga Region State University of Physical Culture, Sport and Tourism, Kazan, 420138 Russia; 8 Laboratory of Genetics of Aging and Longevity, Kazan State Medical University, Kazan, 420012 Russia; 9 Department of Physical Education, Plekhanov Russian University of Economics, Moscow, 115093 Russia; 10 Research Institute for Sport and Exercise Sciences, Liverpool John Moores University, Liverpool, L3 5AF UK; 11 Sports Science Faculty, Lokman Hekim University, Ankara, 06510 Türkiye

**Keywords:** Athletic performance, Caffeine, Coffee consumption, Exercise, Metabolism, Supplements

## Abstract

**Background:**

This study aimed to achieve a dual objective: to compare the frequencies of *CYP1A2* rs762551 genotypes between team sport athletes and a control group, and to determine the association between the rs762551 polymorphism and changes in physical performance after a six-week training program among elite basketball players.

**Methods:**

The study encompassed an analysis of 504 individuals, comprising 320 athletes and 184 controls. For the Turkish cohort, DNA was isolated using the buccal swab method, and genotyping was conducted using the KASP technique. Performance assessments included the Yo-Yo IR2 and 30 m sprint tests. For Russian participants, DNA samples were extracted from peripheral blood, a commercial kit was used for DNA extraction, and genotyping of the rs762551 polymorphism was conducted using DNA microarray.

**Result:**

Notably, a statistically significant linear decline in the prevalence of the CC genotype was observed with ascending levels of athletic achievement within team sports (sub-elite: 18.0%, elite: 8.2%, highly elite: 0%; *p* = 0.001). Additionally, the CA genotype was the most prevalent genotype in the highly elite group compared to controls (80.0% vs. 45.1%, *p* = 0.048). Furthermore, statistically significant improvements in Yo-Yo IR2 performance were noted exclusively among basketball players harboring the CA genotype (*p* = 0.048).

**Conclusions:**

The study’s findings indicate that the rs762551 CC genotype is a disadvantage in elite team sports, whereas the CA genotype provides an advantage in basketball performance.

## Introduction

Caffeine is a widely used stimulant in team sports due to its physiological effects on adenosine receptors in the central nervous system (CNS) [1, 2]. It blocks the downregulation of neuronal activity as an antagonist of the adenosine receptor in the CNS [3, 4], resulting in a series of physiological reactions including neurotransmitter release, increase in muscle activity and induction of adrenalin secretion [5–7], which causes the elevated focus and resistance and a decrease in cognitive effort and pain [7]. Thus, caffeine uptake has been underlined to affect athletic performance via a positive ergogenic impact, bringing about increased effort levels and individual differences [8]. Moreover, caffeine has definite influences on both aerobic (endurance-oriented sporting disciplines) and anaerobic (power-oriented sporting disciplines) metabolism [9–11]. Furthermore, caffeine may influence training-induced changes in physical performance [12, 13]; nevertheless, the observed changes may be individual- and study-dependent [14]. Factors including environmental conditions, training type, sporting experience, and caffeine quantity and timing may alter the individual response to caffeine. Importantly, the genetic background has also been reported to play a role in the response to supplements [15–17]. Considering that diet may affect the cellular response in the gene expression levels [8, 18], the involvement of the genetic diversity between athletes may be critical in examining the effect of caffeine.

Studies in this emerging academic field, which seeks to correlate genetic variations with athletic parameters in athletes, are grouped under the umbrella of sports genetics. In sports genetics research, variations in genes involved in muscle development, cardiovascular pathways, and energy metabolism have been identified in elite athletes from different populations. It has been reported that this information can be used to guide individuals to the appropriate sport discipline at an early age, to determine the suitable types and durations of training for elite athletes, and to address possible injuries with the most accurate methods. Therefore, it should be noted that evaluations based solely on anatomical, physiological, psychological, and/or biochemical data may be insufficient and/or misleading [19]. Particularly after the completion of the Human Genome Project (HGP; 1990–2003), the relationship between genetic variations and athletic performance has garnered increasing attention [20]. Moreover, genetic variations can also be used to decipher possible associations, including diet, in athletes following critical cohort studies. In exercise nutrigenetics, one of the most studied genetic markers, especially in relation to caffeine, is the *CYP1A2* gene rs762551 polymorphism [21].

Cytochrome Cytochrome P450 1A2 (*CYP1A2*), located on chromosome 15 and is approximately 7.8 kb long is a gene involved in the metabolism of caffeine. The gene encodes the primary enzyme responsible for caffeine metabolism, Cytochrome P450 1A2, which is found exclusively in the liver. The polymorphisms in this gene have been underlined to increase the levels of metabolites such as paraxanthine, theobromine, and theophylline, altering the clearance rates of caffeine. These biochemical changes can influence the physiological, metabolic, and exercise effects observed after acute caffeine supplementation [22, 23].

Within the *CYP1A2* gene, a specific single nucleotide polymorphism (SNP), rs762551 (NM_000761.5:c.-9–154 C > A), located in the intron 1, has been associated with the duration of the effects of the caffeine. Individuals with the AA genotype metabolize caffeine quickly and are considered fast metabolizers. They exhibit higher *CYP1A2* enzymatic activity, leading to rapid caffeine clearance from the body. However, the presence of the C allele results in slower metabolism. The individuals with CA genotype are regarded to be intermediate metabolizers whereas the individuals with the CC genotype are slow metabolizers, having lower *CYP1A2* enzyme activity, resulting in slower caffeine clearance [22]. Although not entirely consistent across studies, evidence suggests that slow caffeine metabolizers may not reap the same benefits of caffeine intake on aerobic, strength, and cognitive performance as fast metabolizers do. However, the existing literature on this topic is limited, indicating a need for further research. Consequently, it could be hypothesized that the frequency of the *CYP1A2* CC genotype might be lower among elite athletes compared to their less skilled counterparts due to the potential adverse effects of slow caffeine metabolism [24–26].

From this point of view, the objectives of this research were (i) to compare the frequencies of the *CYP1A2* rs762551 genotype among team sport athletes with those of a control group and (ii) to investigate the potential link between rs762551 and changes in physical performance following a six-week training program, specifically targeting elite basketball players. The hypothesis was that individuals with AA/CA genotypes would show a superior and more effective response to exercise stimuli, resulting in enhanced physical performance.

## Materials and methods

### Ethical approval

The research endeavor adhered rigorously to the principles and ethical guidelines outlined in the Declaration of Helsinki. Ethical clearance and authorization were diligently secured through the Tetowa University Ethics Committee, as evidenced by their official decision number (27/05/2022-27/05/2023). Furthermore, ethical approval was also diligently obtained from the Ethics Committee of the Federal Research and Clinical Center of Physical-Chemical Medicine of the Federal Medical and Biological Agency of Russia, bearing the approval number 2017/04. These meticulous steps underscore the ethical integrity and robustness of the study’s research design and conduct.

### Participants

The study encompassed a comprehensive cohort of 504 individuals, meticulously divided into two distinct groups, comprising 320 individuals in the athlete group and 184 individuals in the control group. The participants were sourced from both Türkiye and Russia, ensuring a diverse representation. Specifically, within the Turkish cohort, a subset of 25 professional male basketball players and an equivalent number of male controls, characterized as physically inactive students, were thoughtfully recruited. The recruitment of all participants transpired over a specific timeframe, spanning from November 12, 2022, to January 08, 2023. The mean age of these participants was calculated to be 25.2 years, with a standard deviation of 2.8 years, thus providing insight into the age distribution within the study population. It is imperative to note that the control group, although devoid of structured training, underwent performance assessments twice over the 6-week study duration. Furthermore, participants in both groups were conscientiously advised to restrict their caffeine intake, primarily through coffee consumption, immediately preceding the scheduled measurement sessions. This precautionary measure aimed to mitigate potential confounding effects associated with caffeine on the study’s outcomes and ensured that the assessments accurately reflected the participants’ inherent physical capabilities.

The research examination encompassed a comprehensive sample size originating from Russia, comprising a total of 295 team sport athletes (*n* = 151 females, *n* = 144 males; 26.3 ± 4.0 years). These athletes were actively engaged in various sporting disciplines, including badminton (*n* = 13), basketball (*n* = 51), baseball (*n* = 23), water polo (*n* = 16), volleyball (*n* = 52), beach volleyball (*n* = 10), handball (*n* = 24), table tennis (*n* = 10), rugby (*n* = 45), softball (*n* = 29), and ice hockey (*n* = 22). The Russian control group consisted of 159 sedentary individuals (*n* = 37 females and *n* = 122 males; 40.3 ± 4.2 years). The inclusion of both genders and the age diversity within the control group aimed to ensure a representative and comparative framework for the research, facilitating a comprehensive analysis of the impact of physical activity and sporting engagement on the study parameters.

The entirety of the athlete cohort, consisting of 320 individuals, underwent a systematic classification based on their athletic achievements and participation levels. This classification stratified the athletes into three distinct categories:

#### Sub-elite (*n* = 78)

This subgroup comprised individuals who demonstrated a high level of proficiency in their respective sports, as they competed in the highest domestic leagues within their country. Notably, these athletes lacked prior experience in international competitions.

#### Elite (*n* = 232)

The elite category encompassed athletes who excelled in their respective sports by competing at the highest domestic league level within their country. Importantly, this subset of athletes had also actively participated in international competitions, thereby showcasing their prowess on a global stage.

#### Highly Elite (*n* = 10)

This select subgroup was composed of exceptionally accomplished athletes who had achieved the pinnacle of success, notably earning the prestigious title of Olympic champions. Their exceptional achievements underscored their unparalleled prowess and dominance in their respective sports disciplines.

This systematic classification scheme facilitated a nuanced examination of the athlete cohort, allowing for the differentiation of individuals based on their level of sporting achievements and international exposure, thereby contributing to a more granular analysis of the research outcomes [27].

### Training and performance tests

The professional basketball players adhered to a rigorous and well-structured training regimen, which took place six days a week, with each session lasting between 60 and 75 min. These training macrocycles were meticulously crafted to provide a comprehensive approach, integrating technical and tactical training activities, league matches, and an intensive program consisting of eight weekly training units. The focus was on enhancing power, speed, and core stability through various drills and exercises, including ball handling, passing, shooting, team tactical drills (offensive and defensive schemes), endurance training, interval training, plyometrics, rebounding and blocking, free throw practice, offensive drills (such as pick and roll, and fast breaks), game simulations, strategy drills, individual technical skill development, team practice, and game sets.

Yo-Yo Test Pro application was used to evaluate the professional basketball players performance. It is noteworthy that the players were already intimately familiar with the test protocols employed in the present study. This familiarity was a direct result of the systematic incorporation of these assessments into the clubs’ monitoring strategy, underscoring their routine utilization as a means of gauging the athletes’ performance and physical condition. Prior to each testing session, a standardized 5-minute warm-up routine was administered. This warm-up protocol comprised a combination of stretching exercises, spanning a duration of 5 to 10 min, and dynamic actions aimed at optimizing the participants’ physical readiness for the ensuing assessments.

Yo-Yo Intermittent Recovery 2 (Yo-Yo IR2) test was selected as the principal method for assessing the participants’ aerobic and anaerobic performance, along with their recovery capacity during intermittent exercise, in accordance with the methodology. This particular test enjoys widespread adoption in the context of team sports due to its high specificity and practical utility. The Yo-Yo IR2 test is structured as follows: Participants engage in a series of 2 × 20 m shuttle runs performed at progressively increasing speeds, each running bout is separated by a brief 10-second recovery interval, and the test is deemed complete when a participant fails to reach the prescribed finish line within the allocated time frame for three consecutive instances [28].

In addition to the Yo-Yo IR2 test, physical performance was further assessed through the execution of the 30-meter sprint test. The administration of these tests adhered to precise and standardized protocols, employing a photosensor system known as the Witty Speed, which is part of the Microgate Equipment suite (Bolzano, Italy). These assessments were conducted on an enclosed running track, spesifically chosen to mitigate the potential influence of adverse weather conditions. The 30-meter sprint test entailed participants commencing from a stationary position placed 0.5 m behind the designated starting line. Each participant completed two successive 30-meter sprints with a 5-minute intermission provided between each attempt. The recorded data encompassed the participants’ fastest sprint times, which were subsequently subjected to detailed analysis.

### Genotyping

Genotyping procedures for the Turkish cohort were meticulously executed in compliance with rigorous standards at the Medical Genetics Laboratory in Ankara, Türkiye. To delineate the genotypic variations, the buccal swab method was employed for DNA isolation, followed by the application of the Kompetitive Allel Specific PCR (KASP) technique. The DNA isolation process was executed utilizing the Buccalyse DNA Extraction Kit, manufactured by Isohelix in the United Kingdom, following the precise guidelines stipulated in the manufacturer’s protocol. Subsequently, the concentrations of the isolated DNAs were ascertained through the utilization of a NanoDrop spectrophotometer, an instrument produced by Thermo Fisher Scientific in the United States. The KASP genotyping method was conducted with attention to details, employing three assay-specific non-labelled oligos. These oligos consisted of two allele-specific forward primers and one common reverse primer, skillfully combined with the KASP Master mix, sourced from LGC Genomics in the United States. The subsequent analysis phase was performed using the 7500 Real-time PCR System, a product of Applied Biosystems in the United States. The analysis culminated in an end-point fluorescent read and comprehensive data analysis, as elucidated in reference [29].

The molecular genetic analysis conducted on the Russian participants involved the utilization of DNA samples that were extracted from peripheral blood specimens. Specifically, venous blood samples, each amounting to 4 milliliters, were carefully collected and deposited into blood collection tubes containing EDTA, using Vacuette EDTA tubes procured from Greiner Bio-One, located in Kremsmunster, Austria. After blood sample collection, the process of DNA extraction and purification was diligently executed following the instructions provided by a commercial kit sourced from Technoclon, based in Moscow, Russia. This procedure ensured the isolation of high-quality DNA from the blood samples, a crucial step in the genotyping process. For the genotyping of the *CYP1A2* rs762551 polymorphism, the microarray analysis technique was adopted. This analysis was conducted using HumanOmniExpressBeadChips, a product developed by Illumina Inc., headquartered in San Diego, California, USA. The genotyping procedure adhered to established methodologies, as previously documented in reference [30].

### Statistical analyses

The statistical analysis was conducted using the Statistical Package for Social Sciences (SPSS) version 25.0. Descriptive statistics, including percentages and means along with their respective standard deviations, were employed to summarize and present the data. Prior to conducting inferential statistics, the assumption of normality was assessed using Skewness and Kurtosis tests. Genotype and allele frequencies were calculated while adhering to the principles of the Hardy-Weinberg equilibrium (HWE), and statistical comparisons were made using χ² and Fisher’s exact tests as appropriate. The paired t-test was applied to assess differences in pre- and post-test performance within the athlete groups, whereas the independent t-test was utilized to investigate variations among groups based on the variables of interest. Hypotheses were tested at a significance level of *p* < 0.05, ensuring a 95% confidence interval. Additionally, to assess the frequency of the CC genotype across the three distinct athlete groups categorized by their levels of achievement, a χ² test for linear trend was employed. These robust statistical methodologies were employed to rigorously analyze and interpret the research findings, ensuring the validity and reliability of the study’s outcomes.

## Results

### Case-control study

The study revealed that there were no significant disparities in allelic frequencies observed between the control groups from Türkiye and Russia, with the A allele prevalence at 62.0% in Turkish and 67.9% in Russian participants, as well as among the athlete groups in which the A allele frequency was 62.0% and 65.8% in Turkish and Russian participants, respectively. Moreover, it is noteworthy that the *CYP1A2* gene rs762551 polymorphism was found to conform to the expectations of the Hardy-Weinberg equilibrium both within the cohort of 320 team sport athletes (*p* = 0.202) and the control participants numbering 184 (*p* = 0.76). This adherence to the Hardy-Weinberg equilibrium suggests that the genetic distribution within the study population was consistent with theoretical expectations. Significantly, a compelling linear trend was discerned in the prevalence of the CC genotype among team sport athletes, wherein there was a decrease in the frequency of this genotype with ascending levels of athletic achievement. Specifically, the CC genotype was observed in 18.0% of sub-elite athletes, 8.2% of elite athletes, and none among the highly elite Olympic champions (0%) (Table 1). This observed linear trend was statistically significant (*p* = 0.001), underscoring a potential relationship between the CC genotype and athletic success. Furthermore, the study disclosed that the frequency of the A allele exhibited a noteworthy difference between elite athletes and sub-elite athletes, with elite athletes demonstrating a higher prevalence of the A allele (68.1% vs. 58.3%). The odds ratio (OR) was 1.5, and the difference was statistically significant (*p* = 0.032), indicating a potential association between the A allele and elite athletic status. Additionally, Olympic champions displayed a significantly elevated frequency of the CA genotype in comparison to control participants (80.0% vs. 45.1%), with an OR of 4.9 and a *p*-value of 0.048. This observation suggests that the CA genotype may be associated with exceptional athletic achievement at the highest level of competition.

**Table 1:** Comparison of genotype and A allele frequencies of rs762551 polymorphism in team sport athletes and control participants

Group	*n*	Genotypes			CC, %	A allele, %
		AA	CA	CC		
Sub-elite athletes	78	27	37	14	18.0	58.3
Elite athletes	232	103	110	19	8.2	68.1*
Highly elite athletes	10	2	8	0	0	60.0
All athletes	320	132	155	33	10.3	65.5
Control group	184	82	83	19	10.3	67.1

^*^p < 0.05, statistically significant differences in the frequency of the A allele between elite and sub-elite athletes

### Training response study

Table 2 reveals compelling insights into the study’s findings regarding athletic performance, particularly in relation to the Yo-Yo IR2 and the genetic influence of the CA genotype. These observations are summarized as follows: Among basketball players, a statistically significant difference in Yo-Yo IR2 performance was discerned when comparing pre- and post-test results (*p* = 0.048) within individuals possessing the CA genotype. Similarly, among the control group, there was a statistically significant difference in Yo-Yo IR2 performance (*p* = 0.001) when comparing pre- and post-test results for individuals with the CA genotype. In contrast, Table 3 elucidates the outcomes pertaining to 30-meter sprint performances. The results indicate that there were no statistically significant differences detected in pre- and post-test sprint performances within each respective group. This implies that the training intervention or assessment period did not lead to significant changes in sprint performance among the study participants, as evidenced by the lack of statistically significant results in the 30-meter sprint tests.

**Table 2:** Association of rs762551 genotypes with pre- and post-yo-yo IR2 test in team sport athletes and control participants

Group	Variable	Genotype	*n*	M ± SD (m)		*t*	*p*
Athletes	Yo-Yo IR2	AA	7	Pre-test	617.14 ± 142.09	-1.89	0.107
				Post-test	685.71 ± 85.41		
		CA	17	Pre-test	662.35 ± 164.90	-2.14	0.048*
				Post-test	752.94 ± 155.71		
		CC	1	Pre-test	780.00	-	-
				Post-test	760.00		
Control group	Yo-Yo IR2	AA	8	Pre-test	617.50 ± 180.93	-1.71	0.130
				Post-test	682.50 ± 195.50		
		CA	15	Pre-test	502.66 ± 177.42	-4.01	0.001*
				Post-test	594.66 ± 172.62		
		CC	2	Pre-test	480.00 ± 56.56	-11.00	0.058
				Post-test	590.00 ± 42.42		

* *p* < 0.05, statistically significant changes within each genotype group

**Table 3:** Association of rs762551 genotypes with pre- and post-30 m test in team sport athletes and control participants

Group	Variable	Genotype	*n*	M ± SD (s)		*t*	*p*
Athletes	30 m sprint	AA	7	Pre-test	5.00 ± 00.35	0.059	0.955
				Post-test	5.00 ± 00.31		
		CA	17	Pre-test	5.06 ± 00.46	-1.337	0.200
				Post-test	5.12 ± 00.48		
		CC	1	Pre-test	4.81	-	-
				Post-test	4.74		
Control group	30 m sprint	AA	8	Pre-test	5.55 ± 00.40	1.832	0.110
				Post-test	5.41 ± 00.58		
		CA	15	Pre-test	5.43 ± 00.58	-0.040	0.969
				Post-test	5.43 ± 00.56		
		CC	2	Pre-test	5.60 ± 00.04	3.000	0.205
				Post-test	5.58 ± 00.03		

* *p* < 0.05, statistically significant differences between the groups

The comparative analysis between groups, while maintaining fixed pre-and post-test parameters, yielded notable findings regarding the influence of specific genotypes on athletic performance, particularly in the context of the Yo-Yo IR2 and 30-meter sprint tests (Table 4). These observations are summarized as follows: Significant differences were observed in the results for the CA genotype when comparing both the Yo-Yo IR2 and 30-meter sprint tests in terms of pre-and post-test parameters. The pre-test results of the 30-meter sprint test were significantly different for individuals with the AA genotype. Importantly, the post-test results of the 30-meter sprint test exhibited significant differences between athletes and the control group for individuals with the AA genotype (*p* < 0.05).

**Table 4:** Association of rs762551 genotypes with Yo-Yo IR2 and 30 m test between team sport athletes and control participants

Genotype	Variable	Group	*n*		M ± SD (m or s)	*t*	*p*
AA	Yo-Yo IR2	Athletes	7	Pre-test	617.14 ± 142.09	-0.004	0.997
		Control	8		617.50 ± 180.93		
		Athletes	7	Post-test	685.71 ± 85.41	0.040	0.969
		Control	8		682.50 ± 195.50		
CA	Yo-Yo IR2	Athletes	17	Pre-test	662.35 ± 164.90	2.638	0.013*
		Control	15		502.66 ± 177.42		
		Athletes	17	Pos-test	752.94 ± 155.71	2.727	0.011*
		Control	15		594.66 ± 172.62		
CC	Yo-Yo IR2	Athletes	1	Pre-test	780.00	-	-
		Control	2		480.00		
		Athletes	1	Pos-test	760.00	-	-
		Control	2		590.00		
AA	30 m sprint	Athletes	7	Pre-test	5.00 ± 00.35	2.769	0.016*
		Control	8		5.55 ± 00.40		
		Athletes	7	Post-test	5.00 ± 00.31	1.651	0.123
		Control	8		5.41 ± 00.58		
CA	30 m sprint	Athletes	17	Pre-test	5.06 ± 00.53	3.58	0.072*
		Control	15		5.43 ± 00.58		
		Athletes	17	Pos-test	5.12 ± 00.58	3.36	0.143
		Control	15		5.43 ± 00.56		
CC	30 m sprint	Athletes	1	Pre-test	4.81	-	-
		Control	2		5.60		
		Athletes	1	Pos-test	4.74	-	-
		Control	2		5.58		

* *p* < 0.05, statistically significant differences between the groups

## Discussion

Researchers are continually discovering new genes and variants on those genes linked to athletic performance [31]. Among these, the *CYP1A2* rs762551 polymorphism is notable [2]. Caffeine is known for its performance-enhancing effects like improved endurance, increased alertness, reduced effort perception, and enhanced muscle contraction. These effects are influenced by caffeine’s interaction with adenosine receptors in the brain, promoting alertness and reducing pain perception during exercise. Research shows that fast metabolizers (AA) generally benefit more from caffeine than slow metabolizers (CC) [22]. Fast metabolizers quickly clear caffeine, leading to a brief but intense performance boost, and can often tolerate higher doses with fewer side effects. In contrast, slow metabolizers have prolonged elevated caffeine levels, which can cause increased heart rate, anxiety, and gastrointestinal issues, potentially impairing performance. Thus, the optimal caffeine dose for performance varies significantly between genotypes. Understanding an athlete’s *CYP1A2* rs762551 genotype can help personalize caffeine use to maximize benefits and minimize side effects [8, 24, 26, 32, 33].

The findings to the utmost extent of our current understanding, this investigation stands as the inaugural endeavor to substantiate that individual harboring the *CYP1A2* rs762551 CC genotype, associated with a diminished capacity for caffeine metabolism (commonly referred as a “slow caffeine metabolism phenotype”), are conspicuously less prevalent among athletes who have attained elite status in the domain of team sports. One of the key findings of the present study highlighted the frequency of the rs762551 A allele is significantly higher in elite team sport athletes when compared to less successful athletes [34]. *CYP1A2* is a so-called inducible enzyme whose expression and function are dominated by its substrate, and the variants on the gene could affect the inducibility. Importantly, rs762551 polymorphism results in high inducibility form of the *CYP1A2* by a trigger factor, which could increase the activity of the enzyme in the presence of its substrate [2, 26, 33, 35]. Thus, allele C may cause delays in the caffeine catabolism, which would trigger imbalances in the glucose metabolism and accordingly high blood pressure with the possibility of diabetes and heart attacks. Therefore, the athletic performances of the athletes with allele C could negatively be affected whereas allele A may be advantageous for the performances of the athletes. The results of the present study confirm the previous studies showing that slow metabolizers do not efficiently respond to caffeine intake and different types of training [22–24, 36]. Furthermore, according to the UK Biobank, the C allele has been shown to be associated with lower coffee intake (*p* = 1.3e-27), increased risk for hypertension (*p* = 1.2e-35), lower vitamin D levels (*p* = 0.003), lower serum albumin levels (*p* = 0.0000021), lower mean corpuscular volume (*p* = 0.0037) and lower physical activity as measured by the frequency of stair climbing in the last 4-weeks (*p* = 0.0025) [34].

The present study also found that statistically significant changes were observed in the Yo-Yo IR2 performance tests in basketball athletes with the CA genotype only. For the CC genotype, no changes were detected due to the statistical limitations (there was only one basketball athlete with the CC genotype). Therefore, it should be noted that our training response study can only be considered as a pilot study and thus an increase of sample size is needed to draw clear conclusions. Interestingly, in a previous study involving elite basketball athletes, it has been shown that caffeine increased jump height in athletes with the AA genotype only [37].

Additionally, it is noteworthy that the ergogenic influence of caffeine, as evidenced in a ball throwing test, exhibited a heightened magnitude among individuals homozygous for the AA allele playing the sport of handball, in comparison to those carrying the C allele [23]. This outcome substantiates the posited conjecture that individuals classified as fast metabolizers manifest more conspicuous and affirmative reactions to the consumption of caffeine.

A limitation of our study was that there was no information regarding habitual coffee intake in our cohort. We can only assume that rs762551 AA genotype carriers consume more coffee than CC genotype carriers, as many studies have previously highlighted [34, 37]. Caffeine is primarily metabolized by *CYP1A2*, and the genetic variations affecting the expression of the enzyme represent a major determinant of enzyme activity. The rs762551 SNP in the *CYP1A2* gene was found to change enzyme activity, as CC genotype carriers reported the lowest enzyme activity [37]. Therefore, high level of coffee consumption in carriers of the AA genotype may be a consequence of a high caffeine clearance rate and obtaining a more positive effect from coffee intake with less negative health effects [38, 39]. To address these limitations and gain a more comprehensive understanding of the relationship between genetics, caffeine metabolism, coffee consumption, and athletic performance, future research endeavors could incorporate more detailed assessments of participants’ coffee habits and explore a wider range of genetic factors. Additionally, longitudinal studies and meta-analyses may help confirm the causal associations suggested by the current findings and provide a more nuanced perspective on the topic.

The *CYP1A2* gene rs762551 polymorphism is one of the most studied genetic markers in exercise nutrigenetics. For instance, Womack et al. [15] found that caffeine intake reduced 40-km cycling time by a greater magnitude in cyclists with AA genotype when compared to C allele carriers. These findings were replicated by Guest et al. [22], showing that caffeine improved 10-km cycling time in AA genotype carriers only. Considering the power- and strength-related phenotypes, only two studies have reported that the AA genotype was associated with improvements of strength [17] and power [24] performance in response to caffeine supplementation. In another study it has been shown that the CC genotype carriers experienced a 12.8% decrease in handgrip strength following caffeine intake [36]. Regarding cognitive performance, there was evidence that the effect of caffeine was greater for *CYP1A2* AA vs. CA/CC genotype carriers for reaction time during exercise [40]. Interestingly, it has recently been reported that rs762551 polymorphism may affect the optimal caffeine ingestion timing [41]. In terms of aerobic capacity enhancement, the authors have recommended ingesting caffeine 1 h before training for fast metabolizers (these athletes might benefit from higher doses of caffeine intake closer to the time of competition) and 2 h before exercise for slow metabolizers (these individuals may need to consume lower doses or ingest caffeine well before competition to avoid prolonged side effects that could hinder performance).

Nevertheless, significant associations of polymorphisms with caffeine-dependent reactions have not widely been reported. Furthermore, it is worth noting that numerous investigations have failed to establish statistically significant associations between the rs762551 polymorphism and alterations in exercise-related traits induced by caffeine [32, 42–46]. The intricacy of these effects, which encompasses epigenetic modulations affecting CYP1A2 protein activity [47, 48], as well as their influence on the addiction-related mechanisms within the system [49], can introduce a level of contention into the findings of the studies. Consequently, it becomes evident that further comprehensive research endeavors are warranted to elucidate the interplay between *CYP1A2* gene polymorphisms, their impact on ergogenic effects, and the dosage of caffeine administered [7].

The conclusions drawn from the study, particularly regarding the *CYP1A2* gene rs762551 polymorphism and its potential influence on athletic performance, offer valuable insights for practical applications in the realm of nutrition for athletes. Athletes, coaches, and nutritionists can consider integrating genetic information, including the *CYP1A2* genotype, into individualized nutrition plans. For those with the CA genotype, who may experience advantages in endurance-based sports like basketball, tailored dietary strategies can be devised to optimize energy metabolism and recovery. Athletes with the CC genotype may metabolize caffeine more slowly, potentially leading to increased sensitivity. Thus, individuals with this genotype might benefit from adjusting their caffeine intake around training and competition to avoid overstimulation or sleep disturbances. Moreover, for athletes, the timing and composition of pre-training meals or snacks can be adjusted based on genetic profiles. Those with the CA genotype, associated with potential advantages in basketball, may benefit from specific pre-training nutrients or supplements to support endurance and performance. Additionally, tailored post-training or post-competition recovery nutrition plans can be developed, considering genetic factors. Athletes with different genotypes may have varying nutrient needs for muscle recovery and glycogen replenishment.

In the context of the conceptualization, the present study may have several limitations and biases. First of all, the number of Turkish participants was relatively low, making it difficult to draw general conclusions for the population. Secondly, the caffeine intake of the participants was not assessed. Additionally, the results were not evaluated in terms of sex differences due to the high subgrouping for statistical analyses. Finally, a multigenetic approach and co-evaluation of the results with other variants, which may affect the outcomes, were not feasible for the Turkish participants due to the selected methodology. Hence, although the results were supported by the literature, further studies addressing these issues are required.

## Conclusion

In summary, the study’s outcomes shed light on the role of the *CYP1A2* gene rs762551 polymorphism in the context of elite team sports. The rs762551 CC genotype is not a prerequisite for achieving elite status in team sports. It does not appear to be a determining factor for success in this athletic domain. On the other hand, the presence of the CA genotype may confer a distinct advantage in the realm of basketball. Individuals with the CA genotype demonstrated notable performance improvements, as indicated by the Yo-Yo IR2 and 30-meter sprint tests. These findings underscore the potential utility of including *CYP1A2* rs762551 polymorphism analysis alongside assessments of other genetic variations and standard phenotypic evaluations in the talent identification process for team sports. This integrated approach could enhance the accuracy and comprehensiveness of talent identification protocols in this context. However, it is crucial to acknowledge that further research, including replication studies and meta-analyses, is imperative to validate the causal association of the *CYP1A2* rs762551 polymorphism with athletic performance conclusively. Such investigations will help corroborate the observed genetic influences on athletic success and provide a more comprehensive understanding of the complex interplay between genetics and sporting achievement.

## Data Availability

No datasets were generated or analysed during the current study.
